# Generation of One-Dimensional Fibrous Polyethylene Nanocrystals in Epoxy Thermosets

**DOI:** 10.3390/polym14183921

**Published:** 2022-09-19

**Authors:** Honggang Mei, Huaming Wang, Lei Li, Sixun Zheng

**Affiliations:** College of Chemistry and Chemical Engineering and the State Key Laboratory of Metal Matrix Composites, Shanghai Jiao Tong University, Shanghai 200240, China

**Keywords:** epoxy, block copolymer, crystallization-driven self-assembly, nanostructures, toughness improvement

## Abstract

The one-dimensional (1D) polyethylene (PE) nanocrystals were generated in epoxy thermosets via crystallization-driven self-assembly. Toward this end, an ABA triblock copolymer composed of PE midblock and poly(ε-caprolactone) (PCL) endblocks was synthesized via the ring opening metathesis polymerization followed by hydrogenation approach. The nanostructured thermosets were obtained via a two-step curing approach, i.e., the samples were cured first at 80 °C and then at 150 °C. Under this condition, the one-dimensional (1D) fibrous PE microdomains with the lengths up to a couple of micrometers were created in epoxy thermosets. In contrast, only the spherical PE microdomains were generated while the thermosets were cured via a one-step curing at 150 °C. By the use of the triblock copolymer, the generation of 1D fibrous PE nanocrystals is attributable to crystallization-driven self-assembly mechanism whereas that of the spherical PE microdomains follows traditional self-assembly mechanism. Compared to the thermosets containing the spherical PE microdomains, the thermosets containing the 1D fibrous PE nanocrystals displayed quite different thermal and mechanical properties. More importantly, the nanostructured thermosets containing the 1D fibrous PE nanocrystals displayed the fracture toughness much higher than those only containing the spherical PE nanocrystals; the K_IC_ value was even three times as that of control epoxy.

## 1. Introduction

The concept of incorporating block copolymers (BCPs) into thermosetting polymers to create the nanostructures has been widely accepted to obtain the materials with improved thermomechanical properties. Hillmyer et al. [1] first reported the introduction of an amphiphilic diblock copolymer into epoxy thermosets via self-assembly approach. In their protocol, the epoxy precursors acted as the selective solvent of the diblock and self-assembled nanoobjects were created in epoxy precursors. In the presence of the self-assembled structures, the curing reaction was carried out to fix the self-assembled nanostructures. The premise of this approach is that the diblock was self-assembled into the nanodomains in epoxy precursors and then subsequent curing was performed to lock the microphase-separated morphologies. Recently, Zheng et al. [2] found that the self-assembly of blocks prior to curing is not always required. For instance, no self-assembly occurred in the mixtures of epoxy precursors with poly(ε-caprolactone)-*block*-polybutadiene-*block*-poly(ε-caprolactone) triblock copolymer. The microphase-separated morphologies were not generated until the curing reaction was performed at a sufficiently high conversion. In this case, the generation of nanostructures in epoxy thermosets followed so-called reaction-induced microphase separation (RIMPS) mechanism. The generation of nanostructures in thermosets is very significant for the improvement of fracture toughness. It is recognized that the optimization of modifier dispersion and interfacial interactions of the modifier with thermosetting matrix is crucial for the improvement of fracture toughness [3,4]. This case was in marked contrast to the modification of thermosets via traditional reaction-induced phase separation approach [5,6,7,8,9]. For the toughening of thermosets with the nanostructures, several mechanisms such as crack tip blunting with nanoobjects, nanocavitation of nanoobjects to induce matrix shearing bands, debonding of nanoparticles following by matrix deformation (or yielding) and nano-bridging of crack could be involved [3,10,11,12,13,14,15,16,17]. Depending on the morphologies of nanostructures, the materials can display quite different thermomechanical properties [11,12,16]. It is highly of interest to modulate the nanostructures to control the thermomechanical properties of thermosets.

By using BCPs, the generation of microdomains in thermosets is mainly based on the hydrophobic interaction of core-forming blocks. In most of the cases, the microdomains are generated in the form of amorphous nanophases since the BCPs are not crystallizable. If a BCP bears crystallizable core-forming blocks, the crystallization of the block will be involved in the process of self-assembly (or RIMPS), constituting so-called crystallization-driven self-assembly (CDSA) behavior. It has been realized that for the formation of assemblies CDSA displays the seeded growth feature which operates under kinetic control. This case quite resembles living/controlled polymerizations of organic molecular monomers, affording well-defined polymer chains with designed molecular weights. Therefore, CDSA has been used for the preparation of high-order and non-spherical (e.g., 1D or 2D) nanoparticles [18,19,20,21,22,23,24,25,26,27,28,29,30].

In the past years, there has been ample literature to report the creation of nanostructures in thermosets by using BCPs [1,2,10,16,31,32,33,34,35,36,37,38,39,40,41,42,43,44,45]. However, there have been few reports involving CDSA behavior of BCPs. Althogh a few BCPs bearing crystalline subchains were utilized [46,47,48,49], the crystallization of subchains have not coupled with the process of self-assemby or/and RIMPS. Guo et al. [47] first reported the generation of nanostructures in epoxy by using crystalline diblock copolymer composed of polyethylene (PE) and poly(ethylene oxide) (PEO). It was found that the spherical PE microdomains were generation in the thermosets while the PE-b-PEO diblock was a minor compoent. Notably, the diblock had a quite low molecular weight with *M*_n_ = 1400 Da and *f*_PE_ = 50%. By using a high molecular weight triblock bearing PE midblock, Zheng et al. [30] obtained the nanostructured epoxy thermosets containing PE microdomains. Notably, only the spherical PE microdmains were generated in the epoxy thermosets. In both of the above cases, no CDSA occured since the curing reactions were carried out above the melting points of PE blocks. More recently, Zucchi and Schroeder et al. [48,49] reported the generation of cylindrical and/or disk-like PE microdomains in epoxy by the use of a PE-*b*-PEO diblock. Notably, the photo-curing of epoxy was performed at room temperature to obtain the thermosets. Under this circustance, the self-assembly of the diblock copolymer was coupled with the crystallization of the PE block, i.e., CDSA behavior occurred. Notably, the PE-*b*-PEO diblock used also had the low molecular weight with *M*_n_ = 1400 Da and *f*_PE_ = 50%.

In this work, we explored to generate the 1D fibrous PE microdomains in epoxy thermosets via CDSA. For this purpose, a PCL-*b*-PE-*b*-PCL triblock copolymer was first synthesized via the ring-opening metathesis polymerization (ROMP) followed by hydrogenation approach. By controlling the curing reactions at different temperatures, the creation of the nanstructures in the thermosets can be modulated by following CDSA or traditional self-assembly mechanisms. As a result, the nanostructured thermosets displayed quite different morphologies. The purpose of this work is: (i) to demonstrate that the 1D PE microdomains can be created via CDSA approach by controlling the curing at specific temperature and (ii) to investigate the impact of the 1D PE nanocrystals on the thermomechanical properties of the thermosets.

## 2. Experimental

### 2.1. Materials

The epoxy monomer used in this work was diglycidyl ether of bisphenol A (DGEBA) and it had the quoted epoxide equivalent weight of 185–210, supplied by Shanghai Resin Co., Shanghai, China. Methyl-5-norbornene-2,3-dicarboxylic anhydride (MNA) was used as the curing agent, purchased from Adamas Regent Co., Shanghai, China. *Cis*-2-butene-1,4-diol and cyclooctene (COE) were supplied by Alfa Aesar Chemical Co., Shanghai, China. ε-Caprolactone (CL), Grubbs 2nd Catalyst (95%), stannous octoate [Sn(Oct)_2_], *p*-toluenesulfonyl hydrazide, ethyl vinyl ether and 2,4,6-tris(dimethylaminomethyl)phenol (DMP-30) were obtained from Adamas Regent Co., Shanghai, China. Tri-*n*-propylamine was purchased from Meryer Co., Shanghai, China. Before use, CL was purified via distillation under reduced pressure over CaH_2_. Anhydrous toluene was prepared via refluxing over sodium and distillation. The PCL-*b*-PE-*b*-PCL triblock copolymer was synthesized via the ring opening metathesis polymerization of COE followed by hydrogenation approach as detailed in Supporting Information (SI).

### 2.2. Preparation of Nanostructured Thermosets

Typically, DGEBA (4.714 g) and MNA (4.286 g) were mixed at 80 °C and then PCL-*b*-PE-*b*-PCL (1.000 g) dissolved in toluene (10 mL) was added with vigorous stirring. Thereafter, the following two curing cycles were used to obtain the nanostructured thermosets, respectively. First, the mixture was rapidly heated up to 150 °C, at which the solvent was rapidly evaporated. Thereafter, 2,4,6-tris(dimethylaminomethyl)phenol (0.6 wt% with respect of the precursors of epoxy (viz. DGEBA + MNA) was added with vigorous stirring. The mixture was cured at 150 °C for 5 h to obtain the thermosets. Second, the mixture was maintained at 80 °C for 1 h to evaporate the majority of solvent; the residual solvent was eliminated in vacuo at 80 °C for 30 min. Thereafter, 2,4,6-tris(dimethylaminomethyl)phenol (0.6 wt% with respect of the precursors of epoxy was added with vigorous stirring. Keeping at 80 °C, the mixture was cured for 24 h. Thereafter, the mixture was heated up to 150 °C for 5 h to obtain the thermosets.

## 3. Results and Discussion

### 3.1. Synthesis of PCL-b-PE-b-PCL Triblock Copolymer

**Scheme 1:**
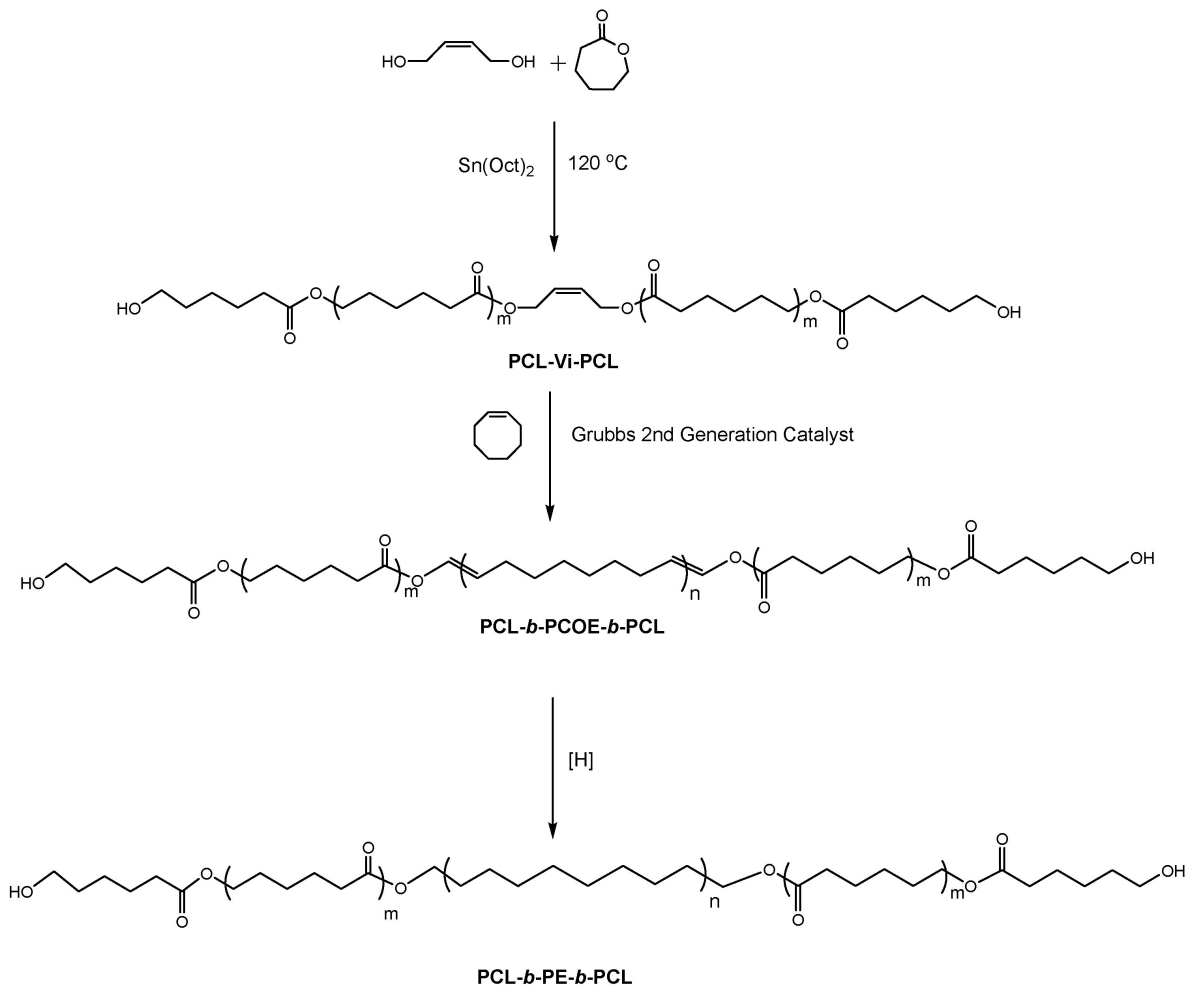
Synthesis of PCL*-b-*PE*-b-*PCL triblock copolymer.

**Figure 1:**
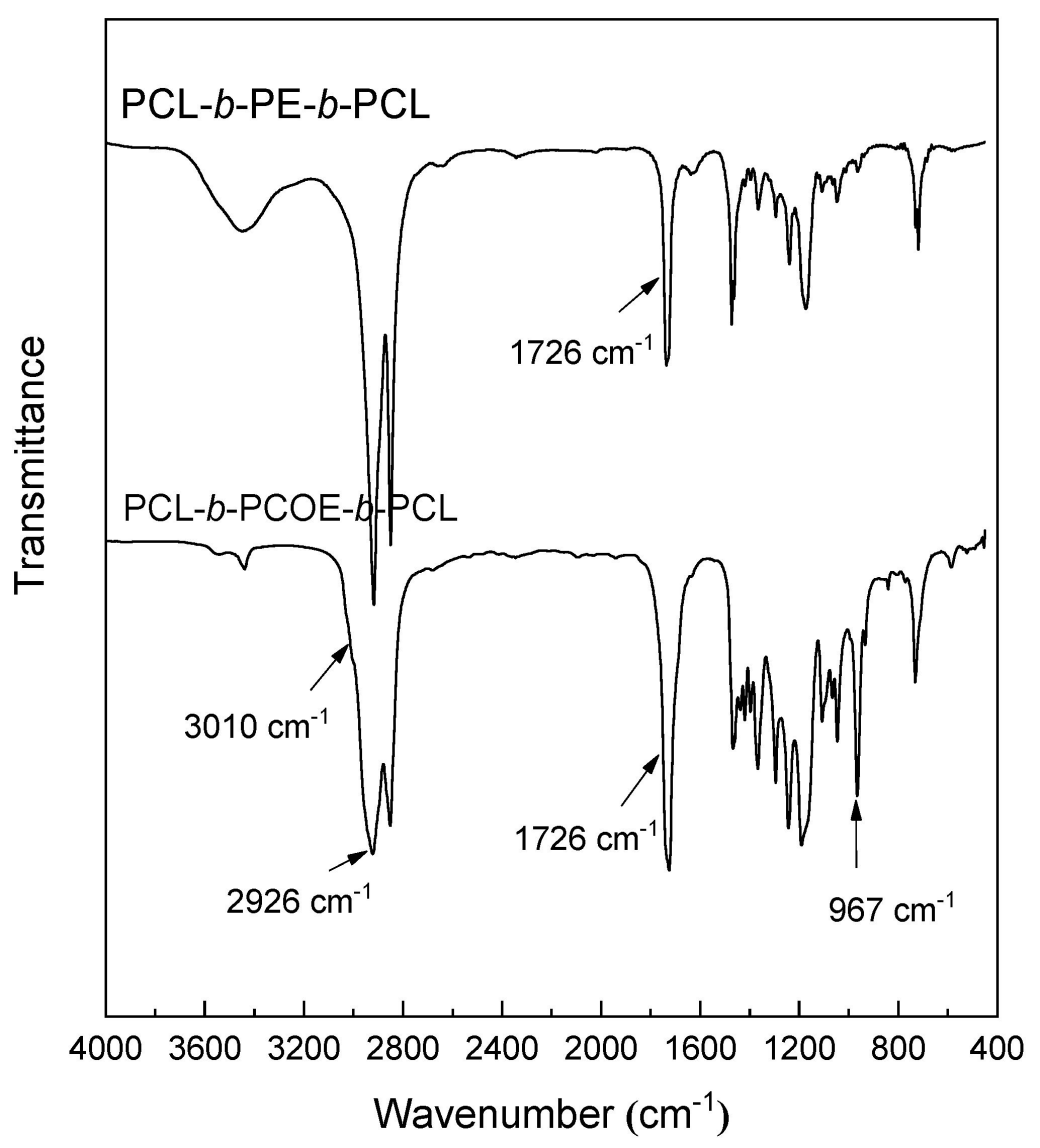
FTIR spectra of triblock copolymers.

**Figure 2:**
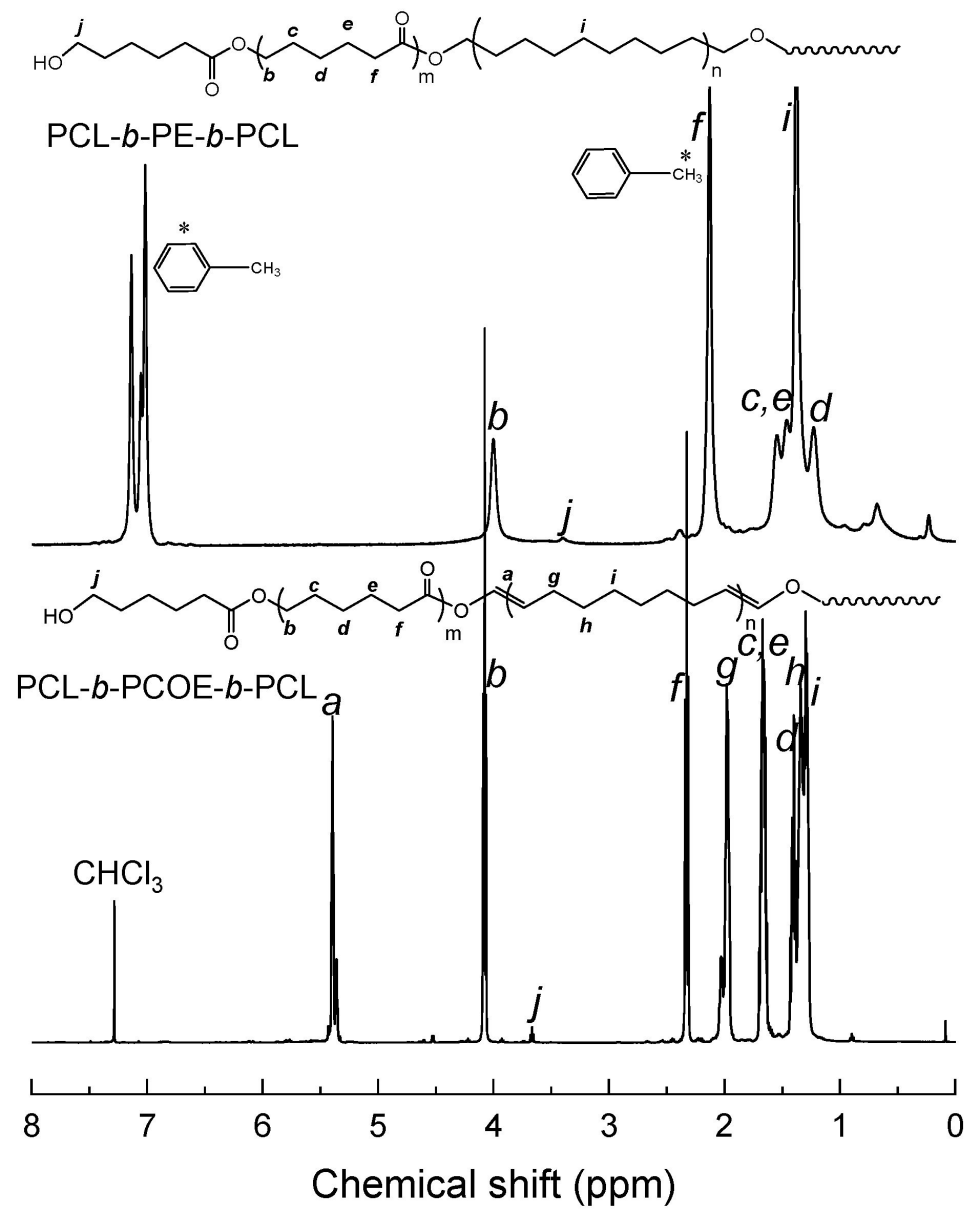
^1^H NMR spectra of triblock copolymers. Asterisk * peaks resulted from deuterium toluene, which was used to dissolve the triblock.

**Figure 3:**
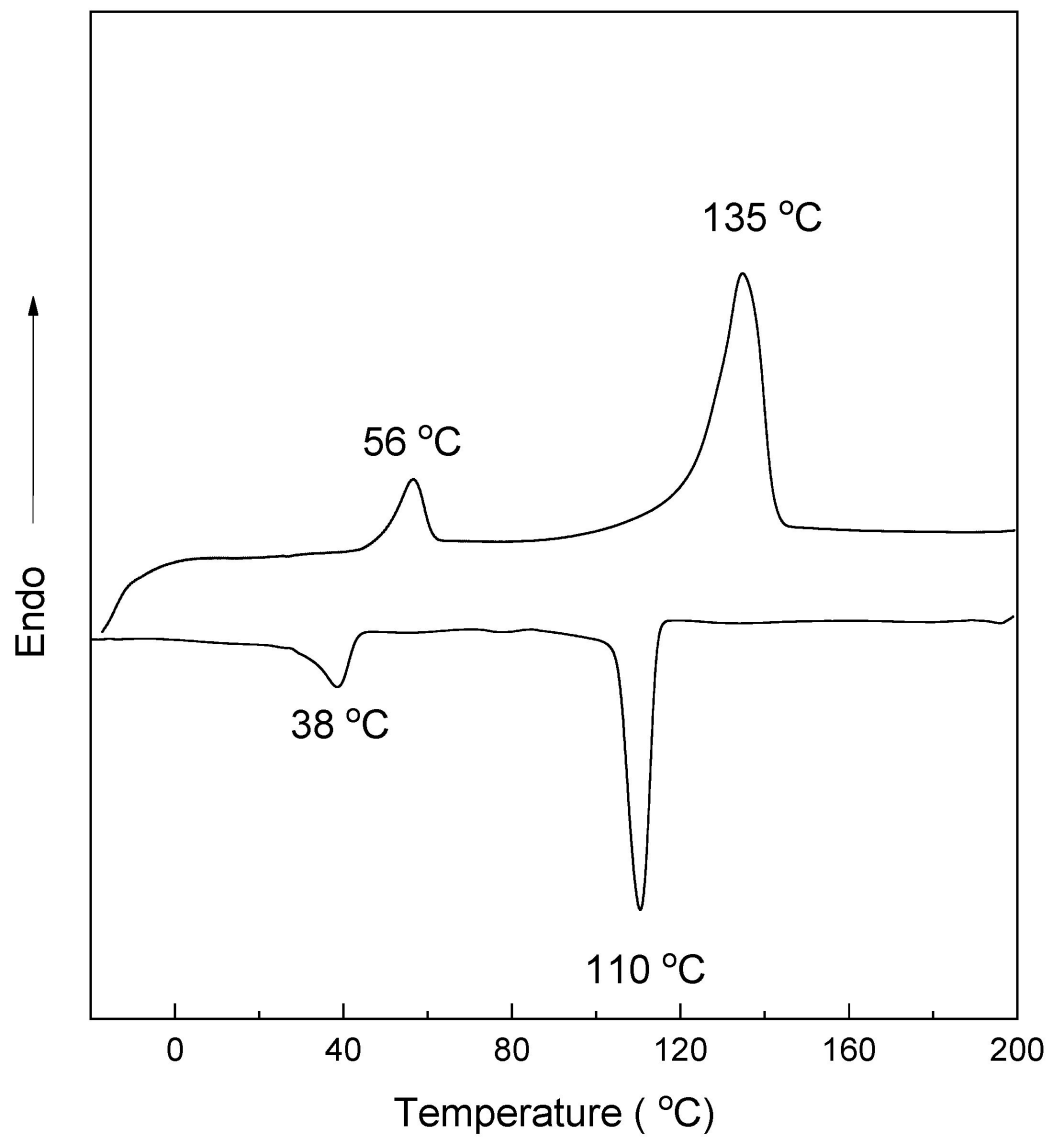
DSC curves of PCL*-b-*PE*-b-*PCL. Up: heating scan; down: cooling scan.

The PCL*-b-*PE*-b-*PCL triblock copolymer was synthesized with a two-step route as shown in Scheme 1. In the first step, a poly(ε-caprolactone)-*block*-polycyclooctene-*block*-poly(ε-caprolactone) (PCL*-b-*PCOE*-b-*PCL) triblock copolymer was synthesized via the ring opening metathesis polymerization (ROMP) of cyclooctene. For the ROMP, the macromolecular chain transfer agent (Macro-CTA) was synthesized through the ring opening polymerization (ROP) of ε-caprolactone (CL) with *cis*-2-butene-1,4-diol as the initiator. The gel permeation chromatography (GPC) showed that the Macro-CTA had the molecular weight of *M*_n_ = 12,100 Da with *M*_w_/*M*_n_ = 1.3 (See Figure S1). The quite narrow distribution of molecular weights indicates that the ROP of CL was successfully performed in a living/controlled fashion. In the second step, PCL*-b-*PCOE*-b-*PCL was hydrogenated into PCL*-b-*PE*-b-*PCL. Shown in Figure 1 and Figure 2 are the FTIR and ^1^H NMR spectra of these two triblock copolymers, respectively. For PCL*-b-*PCOE*-b-*PCL, there was an intense band at 1726 cm^−1^, assignable to the stretching vibration of carbonyl groups of PCL; the bands at 3010 and 967 cm^−1^ are attributable to the stretching and bending vibration of C-H bonds in -HC = CH- moieties, respectively. After hydrogenation, notably, the band at 1726 cm^−1^ remained invariant. Nonetheless, these two bands at 3010 and 967 cm^−1^ disappeared, indicating that the PCOE block was fully hydrogenated into the polyethylene (PE) blocks. As shown in the ^1^H NMR spectra (See Figure 2), the peaks at 4.08, 3.67, 2.33, 1.67 and 1.40 ppm are attributable to the resonance of methylene protons, which are characteristic of the methylene protons of PCL chains. Of these peaks, the peak at 3.67 ppm is assignable to the protons of hydroxymethyl groups at the ends of PCL chains. By using the integral intensity ratio of the peak at 3.67 ppm to that at 4.08 ppm, the lengths of PCL blocks were calculated with the following equation:

*L*_PCL_ = *A*_4_._08_/*A*_3_._67_ × *M*_CL_ × 2 (1)

where *A*_3_._67_ stands for the integral intensity for the peak at 3.67 ppm where *A*_4_._08_ represents for that at 4.08 ppm. *M*_CL_ is the mole mass of CL (*M*_CL_ = 114.14 Da). The lengths of PCL subchains were calculated to be *L*_PCL_ = 10,500 Da. Combined with the GPC results, the length of PCOE was estimated to be *L*_PCOE_ = 11,700 Da. In addition, the hydrogenation of PCOE blocks was demonstrated by the complete shift of peak from 5.39 to 1.38 ppm. The former is attributed to the methine protons of PCOE blocks whereas the latter to the methylene protons of PE blocks. It is worth noticing that the signals of the resonance assignable to PCL methylene protons remained unchanged with the occurrence of hydrogenation. This observation indicates that the PCL endblocks remained less affected, i.e., the hydrogenation only occurred in the PCOE chains. The similar results were also found for the hydrogenation of PCOE in the presence of acrylate moieties as reported by other investigators [50,51,52]. It should be pointed out that the route of synthesis for PCL-*b*-PE-*b*-PCL triblock was different from the ROP approach with a PE diol as the macromolecular initiator [47]. The hydrogenated product was subjected to thermal analysis by means of differential scanning calorimetry (DSC) and the DSC thermograms are shown in Figure 3. The melting and crystallize transitions of PCL endblocks were detected at 56 and 38 °C whereas those of PE midblock occurred 135 and 110 °C, respectively. The fact that the *T*_m_ of PE block was higher than that of commercial PE indicates that the PE block had quite high regularity of chain, i.e., there were few branched structures along the main chain. The above results of structural characterization indicate that the triblock copolymer with PE midblock and PCL endblocks was successfully synthesized.

### 3.2. Preparation of Nanostructured Epoxy Thermosets

**Scheme 2:**
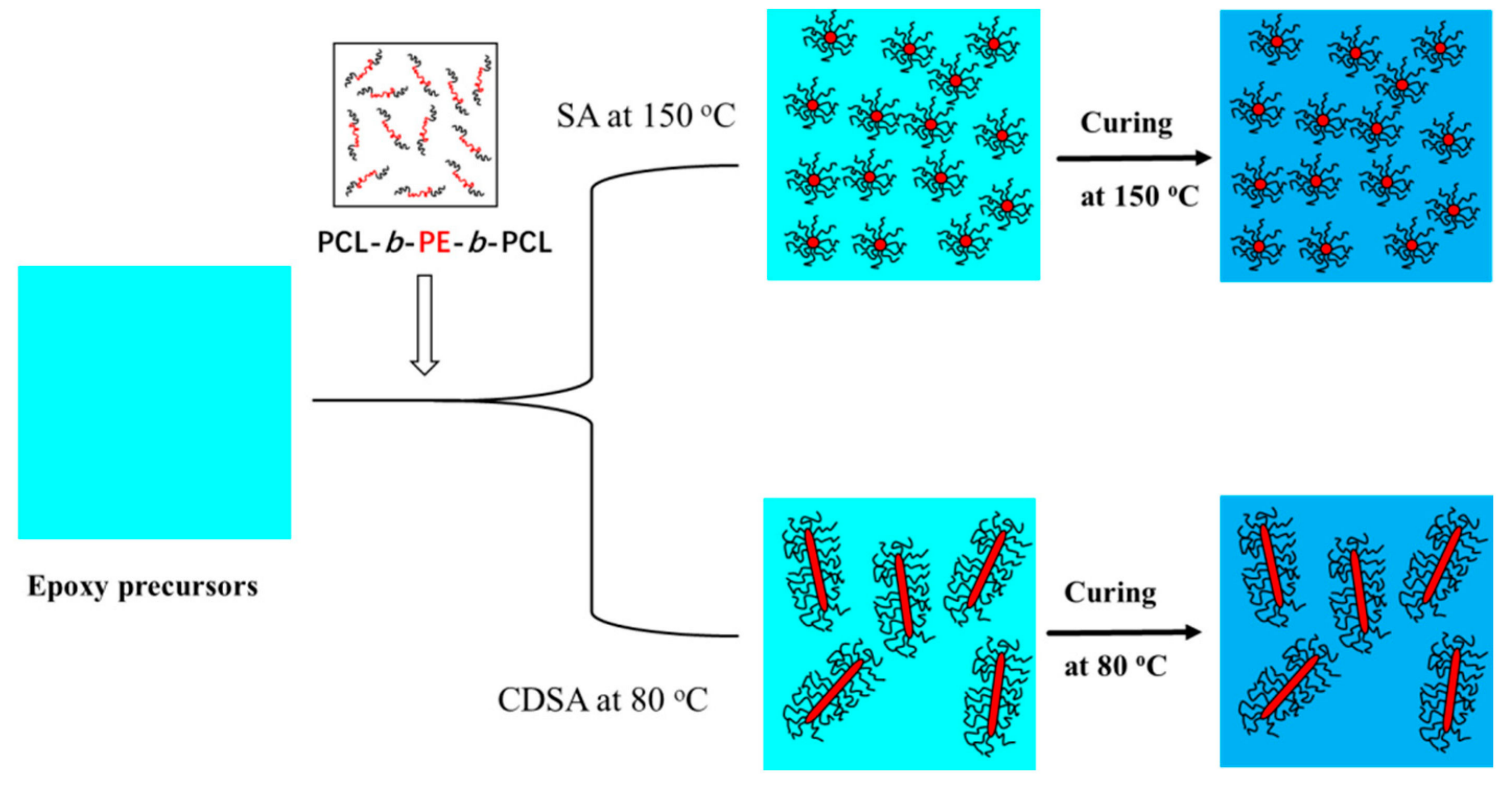
Generation of spherical and fibrous PE microdomains in epoxy thermosets containing PCL*-b-*PE*-b-*PCL triblock copolymer.

In this work, the nanostructured epoxy thermosets were prepared via two different approaches as shown in Scheme 2. In the first approach, the PCL*-b-*PE*-b-*PCL triblock was mixed with the epoxy precursors (viz. DGEBA + MNA) at 150 °C and thereafter the curing reaction was also performed. The purpose to use the curing temperature higher than the melting point of PE (*T*_m_ = 135 °C) is to allow the occurrence of the self-assembly of the triblock in the absence of PE crystallization, i.e., the crystallization of PE did not occur until the curing reaction was undergone completion. Of course, the crystallization occurred while the cured product was cooled to room temperature. Notably, all the thermosets containing PCL-*b*-PE-*b*-PCL triblock were transparent, indicating that no macroscopic phase separation occurred in the curing process. In the second approach, the triblock was first dissolved in a small amount of toluene and then the solution was dropwise added to the epoxy precursors at 80 °C, at which the solvent was slowly evaporated. In addition, at this temperature, the mixtures were slowly cured for a very long time (viz. 24 h). Under this curing condition, notably, the mixtures were also vitrified, and the thermosets were obtained. Notably, the cured thermosetting blends were translucent, especially while the concentration of triblock was 10 wt% or higher, which was in marked contrast to the situation that the samples were cured at 150 °C. To promote the curing reaction to undergo completion, the thermosets were post-cured at 150 °C. Notably, the post curing did not alter the appearance of the thermosets. It should be pointed out that the first-stage curing was carried out at 80 °C, which was much lower than the melting point of PE (*T*_m_ = 135 °C). Therefore, the crystallization of PE blocks would be coupled with the self-assembly of the triblock. It is of interest to examine the morphologies of the thermosetting blends via these two curing approaches.

**Figure 4:**
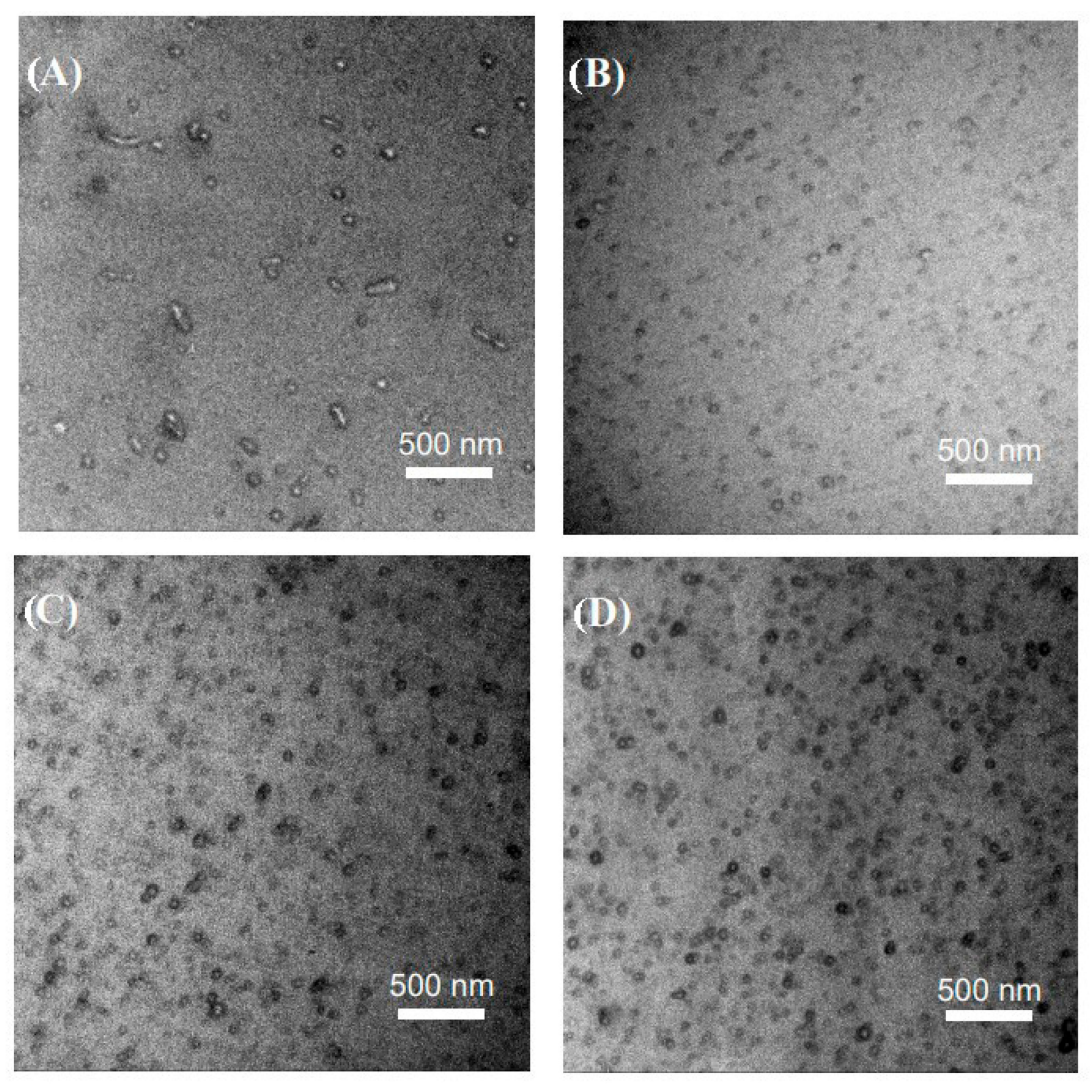
TEM images of epoxy thermosets containing (**A**) 5, (**B**) 10, (**C**) 15 and (**D**) 20 wt% of PCL*-b-*PE*-b-*PCL via SA.

**Figure 5:**
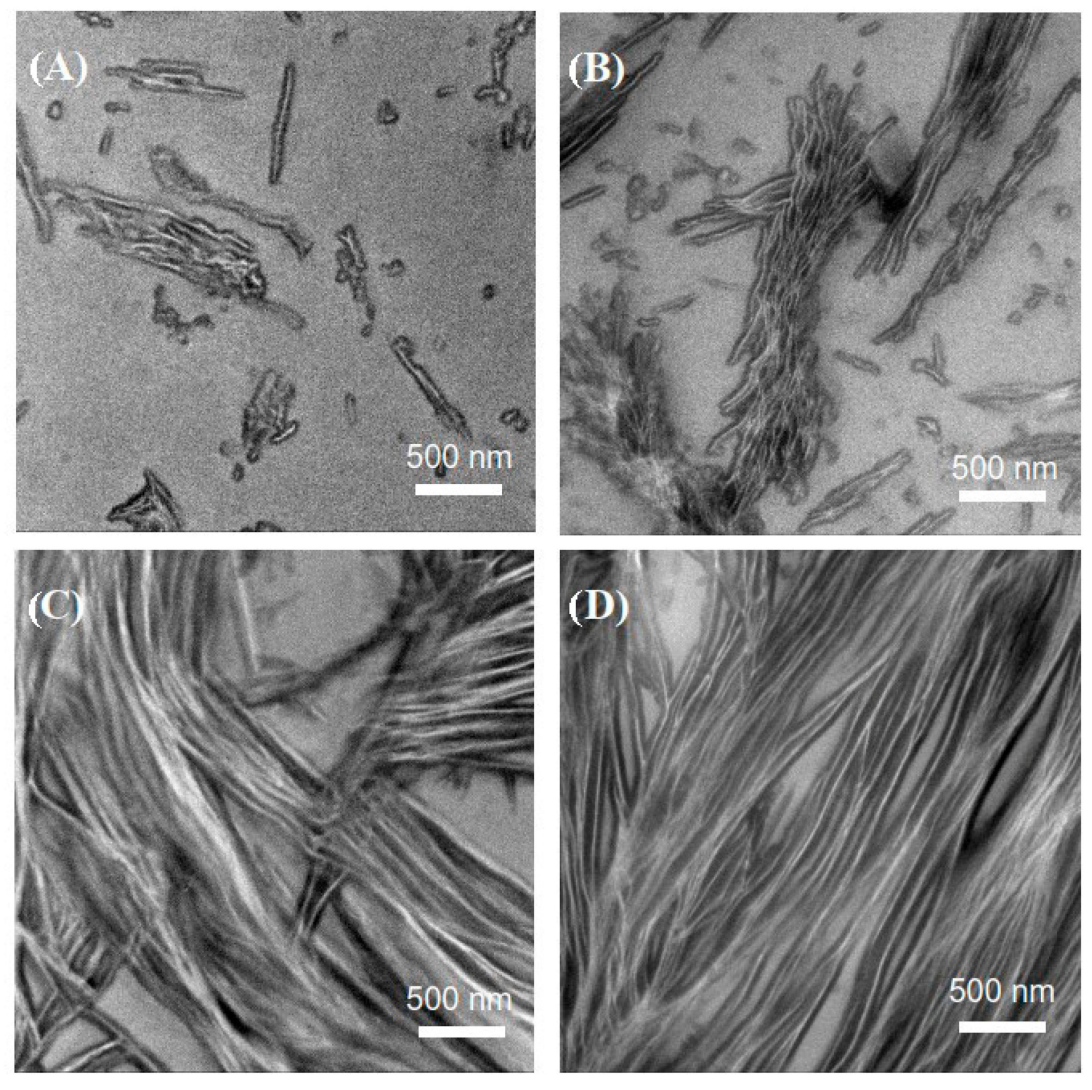
TEM images of epoxy thermosets containing (**A**) 5, (**B**) 10, (**C**) 15 and (**D**) 20 wt% of PCL*-b-*PE*-b-*PCL via CDSA.

The morphologies of the thermosets were investigated by means of transmission electron microscopy (TEM). The samples were sliced with ultrathin microtome and the sections were stained with ruthenium tetraoxide (RuO_4_). Under this circumstance, the epoxy matrix which was mixed with PCL subchains was preferentially stained whereas the PE microdomains remained less affected. Figure 4 and Figure 5 show the TEM images of the epoxy thermosets. For all the samples, the microphase-separated morphologies were exhibited. Considering the difference in electron density, the features surrounded with dark layers are attributable to PE microdomains; the shallow to the epoxy matrix. The dark layer around the PE microdomains could be the PCL chains which were partially demixed out of epoxy matrix in the curing process [1,29]. In all the cases that the samples were prepared with a one-step curing at 150 °C, the spherical microdomains of PE were generated with the size of 20~30 nm in diameter (See Figure 4). The quantity of the PE microdomains increased as the contents of the triblock increased. Notably, the sizes of PE microdomains remained almost unchanged. For the samples which were cured with a two-step approach at 80 °C and then 150 °C, notably, the one-dimensional (1D) fibrous PE microdomains were created. The 1D fibrous PE microcrystals had the lengths up to tens of micrometers and the cross-section diameter of 10~20 nm, depending on the contents of the triblock. The TEM results indicate that: (i) the nanostructures in epoxy thermosets were successfully generated by using PCL*-b-*PE*-b-*PCL triblock; (ii) the morphologies of the nanostructured thermosets were quite dependent on the approaches used for the cure of epoxy.

It is proposed that the generation of nanostructures followed the self-assembly mechanism. In the mixtures composed of the epoxy precursors and the triblock, PCL endblocks was miscible with epoxy whereas the PE midblock was immiscible with epoxy. Therefore, the epoxy precursors can behave as the solvent selective for the PCL blocks and thus the self-assembly occurred prior to the curing reaction and the self-assembled nanostructures can be fixed via curing reactions. Nonetheless, the self-assembly behavior of the triblock copolymer was quite dependent of the temperatures of the mixtures, which were above or below the melting point of PE (*T*_m_ = 135 °C), determining whether the crystallization of PE block occur or not. At 150 °C (i.e., a temperature higher than *T*_m_ of PE), no crystallization of PE occurred and the triblock was self-organized into the spherical microdomains. The microdomains were the micelle-like nanoobjects which were composed of PE cores and PCL coronas which were solvated by the epoxy precursors. The PE microdomains were generated via the hydrophobic interactions of PE blocks and no crystallization was involved. As the curing reaction was performed, the epoxy precursors as well as PCL subchains were gradually crosslinked and finally vitrified. Since the curing temperature (viz. 150 °C) was higher than the melting temperature (*T*_m_) of PE, the molten PE microdomains were fixed and trapped in epoxy matrix. Owing to the restriction of vitrified epoxy matrix, no further aggregation can occur even while the thermosets were cooled to room temperature and there was the decrease in the volumes of PE microdomains which was induced by the crystallization. As a consequence, the spherical PE microdomains were trapped into the epoxy matrices.

At 80 °C (i.e., a temperature lower than *T*_m_ of PE), the triblock was also self-assembled into the spherical microdomains in the epoxy precursors after the removal of the small amount of solvent (viz. toluene). The spherical microdomains were composed of PE core and PCL coronas that were mixed with the epoxy precursors. At the beginning of the self-assembly, the PE cores were amorphous. Nonetheless, the amorphous PE cores would rapidly crystallize at such a low temperature (viz. 80 °C). For a single nascent spherical microdomain, the crystallization would lead to the decrease in the volume of core. As a result, the surface free energy of the microdomain will be rapidly enhanced since the specific surface area was increased. To counteract the increased surface free energy, the nascent spherical microdomains with the crystallized PE cores would have a tendency to aggregate. Nonetheless, the aggregation only occurred along a specific direction until all the spherical micelles were fully consumed since the coronas (viz. PCL subchain) were solvated by the epoxy precursors. The combination of self-assembly with the crystallization constituted a scenario of crystallization-driven self-assembly (CDSA) [24,25,53,54,55]. The CDSA behavior yielded the 1D fibrous nanoobjects as shown in Figure 5. Compared to the curing of epoxy at 80 °C, the CDSA was very fast. Notably, the cure at 80 °C for 24 h afforded the vitrified solids with high rigidity and insolubility in organic solvents such as toluene and tetrahydrofuran. Owing to the occurrence of vitrification, the 1D fibrous PE microdomains were locked in the crosslinked matrix. In this work, a post-curing at 150 °C for 5 h was further applied to the vitrified thermosets, to promote the curing reaction to completion. It is of interest to investigate the impact of the generation of the spherical and fibrous PE microdomains on the thermomechanical properties of the materials (See *infra*).

### 3.3. Crystallization and Melting Behavior

**Figure 6:**
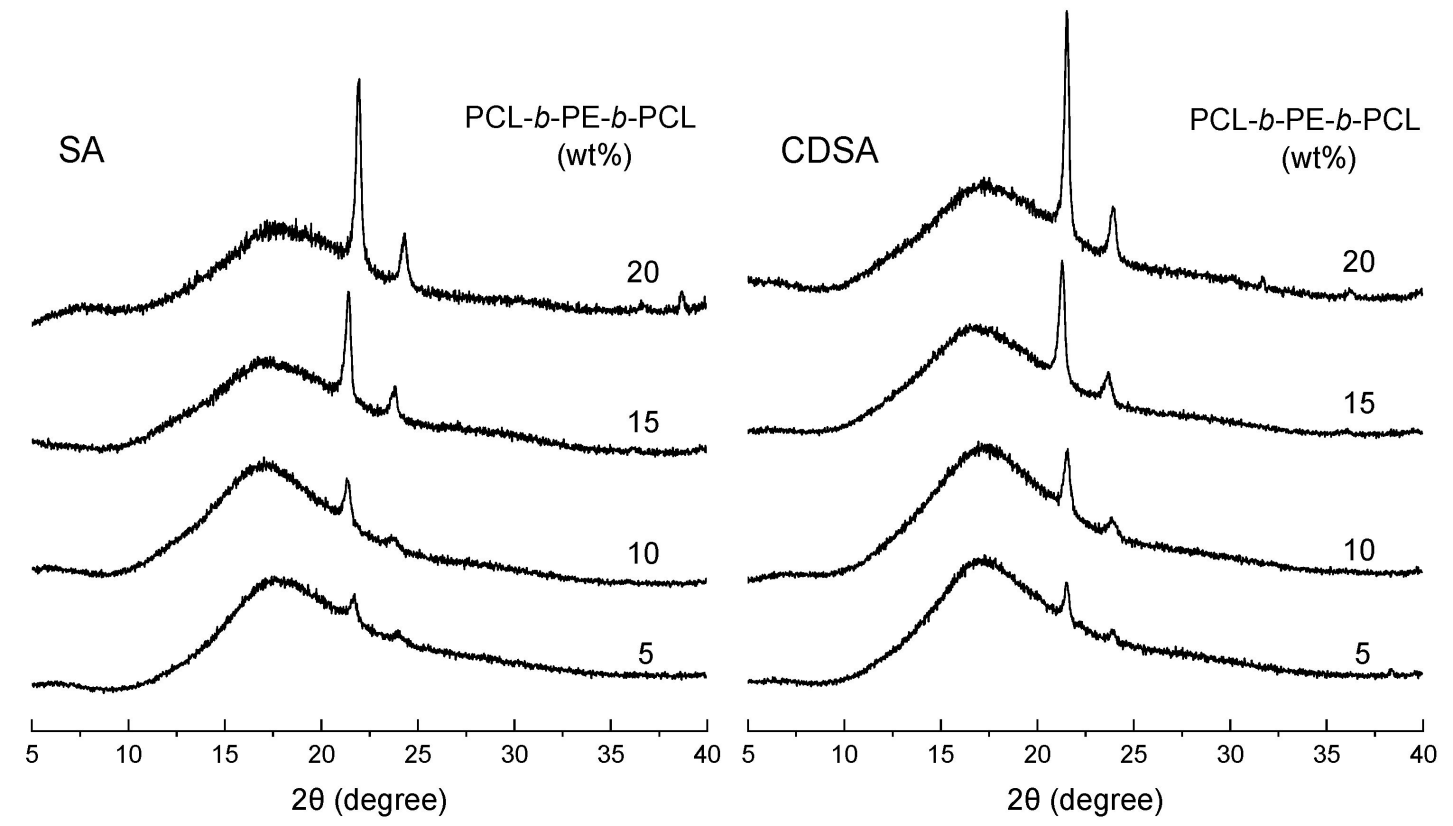
XRD curves of epoxy thermosets containing PCL*-b-*PE*-b-*PCL via SA and CDSA.

The crystalline structures of PE microdomains were studied by means of wide-angle X-ray diffraction (XRD). Figure 6 shows the XRD profiles of the thermosets. In both of the cases, each XRD curve is composed of a broad halo at 2θ = 17.2° and a couple of sharp diffraction peaks. The halos are attributable to epoxy whereas the sharp diffraction peaks to the PE nanocrystals in the thermosets. For the PE nanocrystals, the diffraction peaks were detected at 2θ = 21.92 and 24.33°, responsible for the reflection of (110) and (200) planes in the orthorhombic lattice [56]. For the spherical and fibrous PEO nanocrystals, notably, the positions of diffraction peaks were almost the same, suggesting that in the PE microdomains the crystalline structures of PE were not altered. At the same composition, notably, the intensities of the PE diffraction peaks for the spherical microdomains were slightly lower than those for the fibrous PE nanocrystals, i.e., the crystallinity of the former was slightly lower than that of the latter. The slight difference can be explained on the basis of the behavior of confined crystallization of PE in these two systems. In the microphase-separated thermosets, the PE microdomains were trapped into rigid and crosslinked epoxy matrix, constituting the condition of confined crystallization [47,57]. Depending on the sizes and morphologies of microdomains, the crystallization of PE was restricted at different degrees. The stronger the confinement, the lower the rate of crystallization, the lower the crystallinity. It is proposed that the degrees of confined crystallization in the spherical microdomains were larger than those in the fibrous microdomains and thus could display the lower crystallinity. The XRD results showed that the PE component existed in the epoxy thermosets in the form of the spherical and/or fibrous nanocrystals.

**Figure 7:**
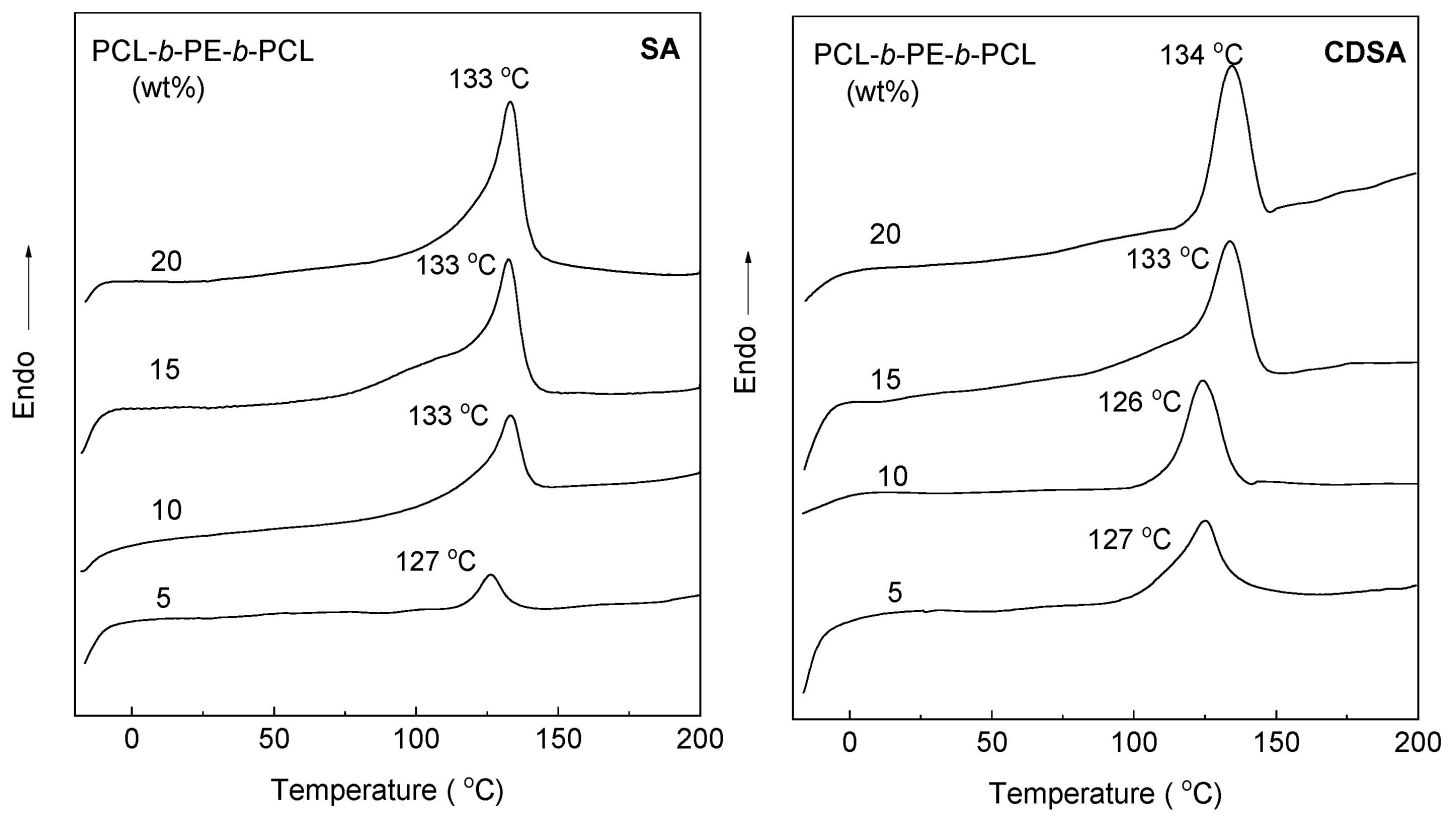
Heating DSC curves of the nanostructured thermosets via SA and CDSA.

**Figure 8:**
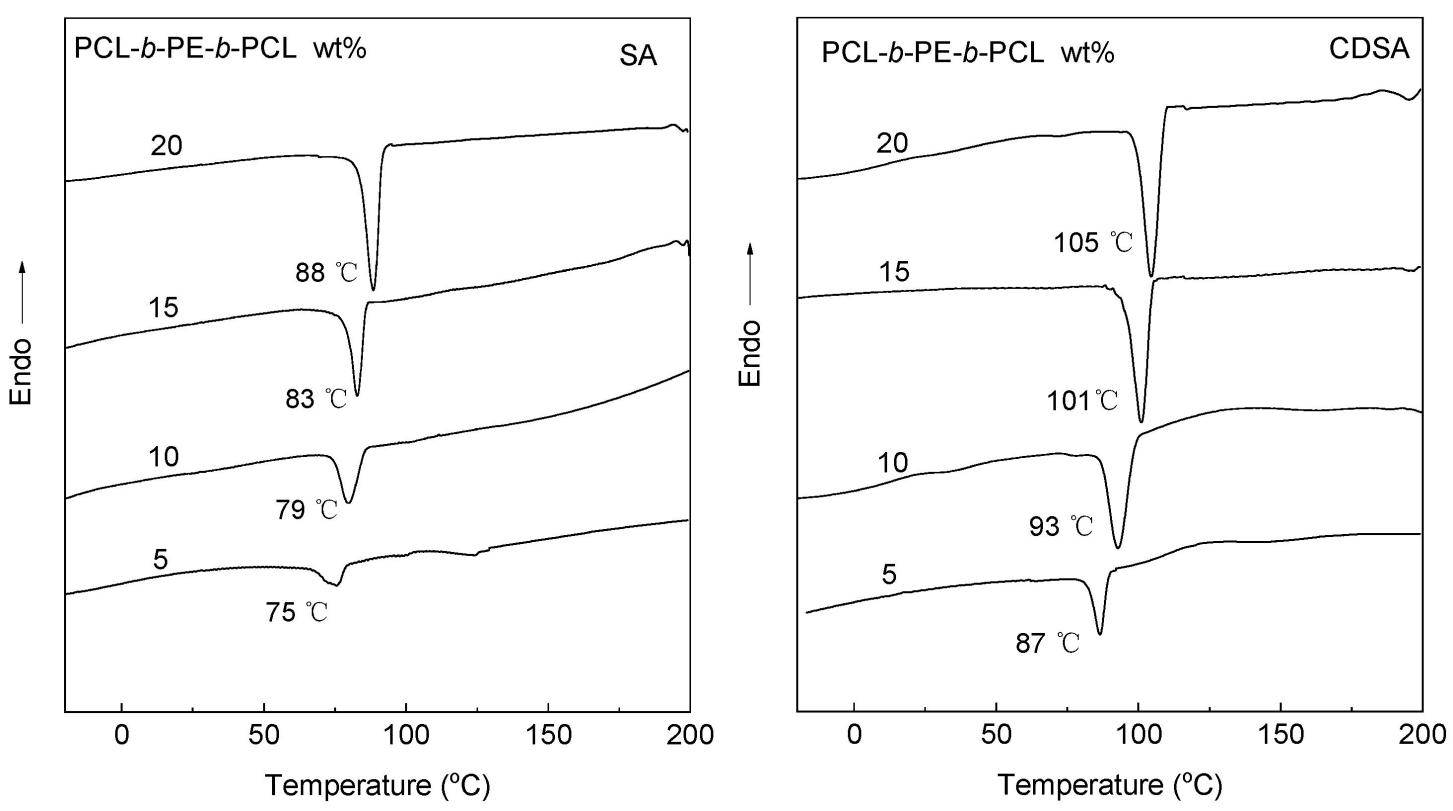
Cooling DSC curves of the nanostructured thermosets via SA and CDSA.

To investigate the melting and crystallization behavior, the nanostructured thermosets containing PE microdomains were subjected to DSC. The heating and cooling DSC scans are shown in Figure 7 and Figure 8. In each heating scan, a single endothermic peak was exhibited at 130~135 °C, the intensity of which increased with the contents of the triblock. The endothermic peaks are assignable to the melting transitions of PE. In each cooling scan, a single exothermic peak was also displayed, assignable to the transition of crystallization while the samples were cooled from melts. In all the cases, no crystallization and melting peaks assignable to PCL subchains were detected, indicating that the PCL subchains were no longer crystallizable in the nanostructured thermosets. This observation is accounted for the miscibility of PCL with the epoxy network. The difference (Δ*T*) between melting temperature (*T*_m_) and crystallization temperature (*T*_c_) is defined as the supercooling, which reflects the driving force for crystallization. The higher the Δ*T* values, the more difficult the crystallization. At the same composition, notably, the Δ*T* values of the thermosets with a one-step curing at 150 °C were much higher than those of the thermosets with a two-step curing at 80 °C and then 150 °C. This phenomenon revealed that the crystallization of PE in the former system required the driving force much higher than in the latter system. In other words, the degrees of confined crystallization in the former system were higher than those in the latter system. Owing to the generation of spherical microdomains (See Figure 4), the crystallization of PE was confined within the spherical spaces in the former system. In the latter system, in contrast, the crystallization of PE was only confined within the 1D fibrous microdomains with the length up to tens of micrometers. Therefore, the confinement of crystallization was significantly diminished. As a result, the crystallization of PE was carried out at the decreased supercooling (Δ*T*).

### 3.4. Dynamic Mechanical Thermal Properties

**Figure 9:**
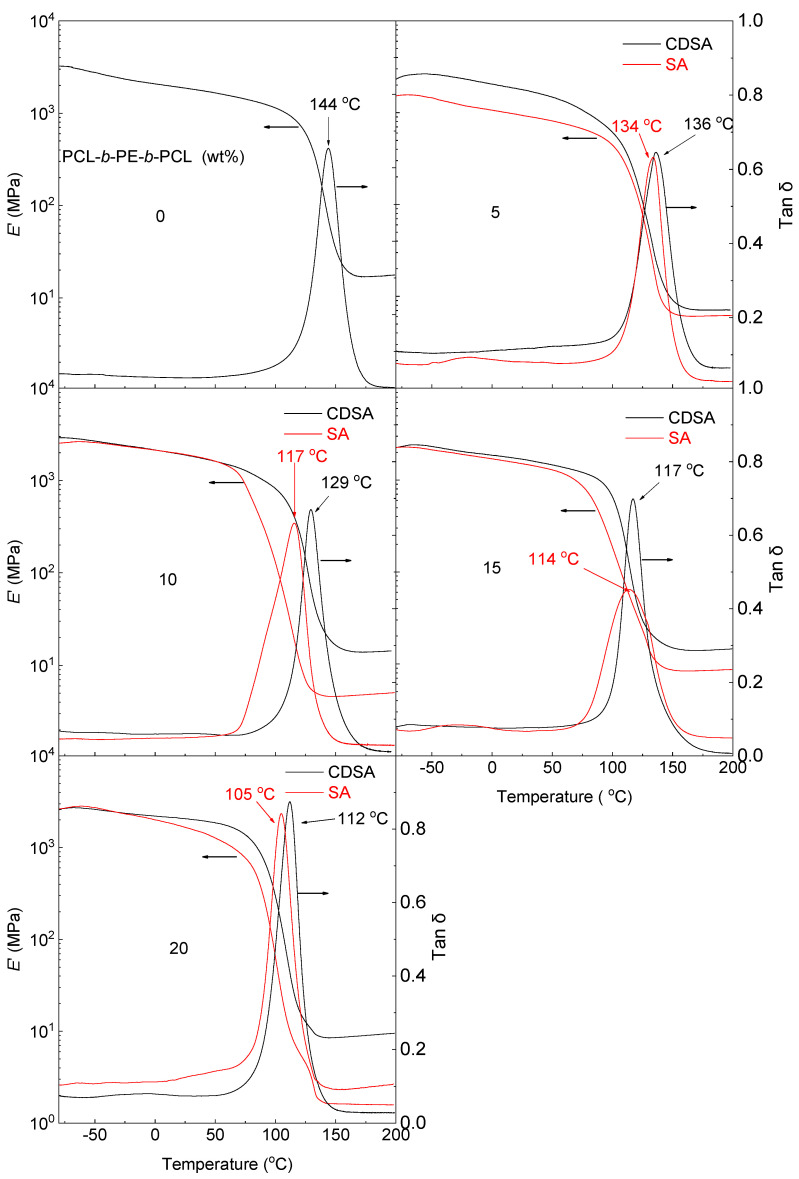
DMTA curves of epoxy thermosets containing 0, 5, 10, 15 and 20 wt% of PCL*-b-*PE*-b-*PCL via SA and CDSA.

**Scheme 3:**
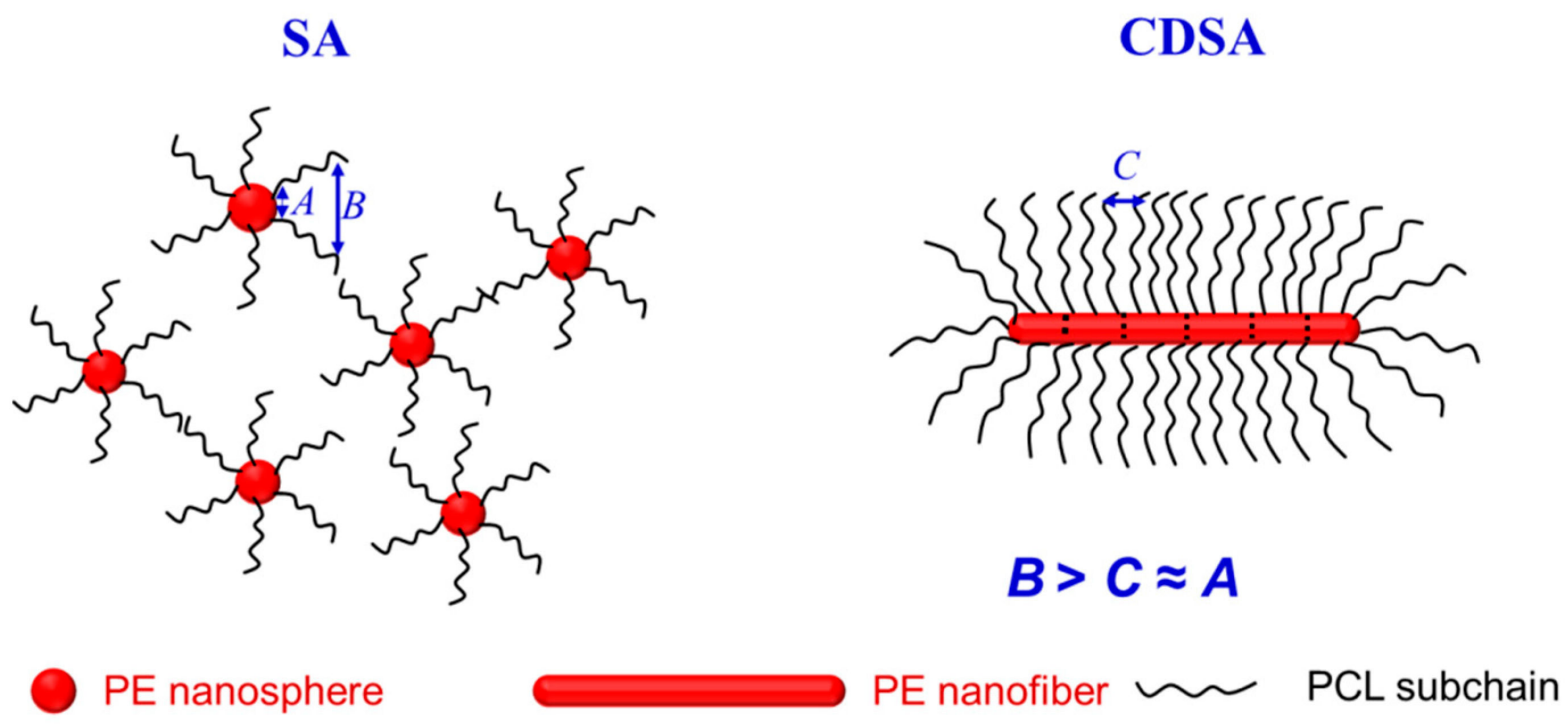
Crowding effect of PCL subchains at the surfaces of spherical and 1D fibrous PE nanocrystals.

The thermomechanical properties were investigated by dynamic mechanical thermal analysis (DMTA). Figure 9 shows the DMTA spectra of the control epoxy and the nanostructured thermosets. For control epoxy, the glass transition temperature (*T*_g_) was detected at 144 °C. Upon introducing the triblock, notably, the *T*_g_’s were decreased. The higher the contents of the triblock; the lower the *T*_g_’s. The decrease in *T*_g_’s is due to the miscibility of the PCL subchains with the epoxy networks. As an epoxy-miscible component, PCL had a quite low *T*_g_ (i.e., *T*_g_ = −65 °C [58]) and can plasticize the epoxy network. Therefore, the *T*_g_’s were significantly decreased. In fact, it is the miscibility that stabilizes the dispersion of the spherical or 1D fibrous PE nanoobjects in epoxy matrix. A further comparison showed that the *T*_g_’s of the nanostructured thermosets via CDSA were significantly higher than those via conventional self-assembly (SA). For instance, when the content of the triblock was 10 wt%, the nanostructured thermoset via CDSA approach had the *T*_g_ = 129 °C, which was much higher than that via SA approach (i.e., *T*_g_ = 117 °C). In the nanostructured thermosets containing block copolymers, the *T*_g_’s of thermosetting matrices were quite dependent on the mixing degrees of the thermoset-philic chains (viz. PCL) with epoxy matrices. Hillmyer and Bates et al. [1,29] have found that in the nanostructured thermosets via self-assembly mechanism, the epoxy-philic subchains were partially demixed out of epoxy matrix. Such a demixing behavior of epoxy-philic subchains has been interpreted on the basis of the occurrence of a transition from equilibrium state to a chemically pinned metastable state while the curing reaction of epoxy progressed through the gel point [1,29]. The demixing degrees of epoxy-philic chains are dependent on: (i) the specific surface area of the self-assembled nanoparticles and (ii) the crowding degrees of epoxy-philic subchains at the surfaces of the self-assembled nanoparticles. The larger the specific surface areas of the self-assembled nanoparticles, the higher the demixing degrees. While the contents of the triblock (or PE subchains) were identical, the spherical PE microdomains had a specific surface area much higher than that of fibrous PE microdomains. If the specific surface area factor were dominant, the demixing degrees of PCL subchains in the system containing the spherical PE microdomains would be higher than those in the system containing the 1D fibrous PE microdomains. Therefore, the *T*_g_’s of epoxy matrices for the former would be higher than those for the latter. Nonetheless, the DMTA results showed that the *T*_g_’s for the latter were higher than those for the former, which is contrary to the judgment on the basis of the specific surface areas of PE microdomains. In fact, the demixing degrees of PCL subchains were additionally affected by the crowding effect of PCL subchains at the surfaces of PE microdomains. It is proposed that at the surfaces of PE microdomains, the PCL chains preferred to take the conformation perpendicular to the surfaces to minimize their mutual crowding. Obviously, the crowding effect is significantly affected by the surface curvature of PE microdomains. The higher the surface curvature of PE microdomains, the smaller the crowding degrees of PCL subchains. Compared to the spherical PE microdomains, the 1D fibrous PE microdomains had the smaller curvature of surfaces and thus the PCL subchains at the surface of the 1D fibrous PE microdomains were more crowded than those at the surface of spherical PE microdomains. For the PCL subchains, the inter-chain distances (i.e., *A* and *C*) intimately at the surfaces of the PE microdomains are quite close for the spherical and 1D fibrous microdomains, which were larger than the inter-chain distance (i.e., *B*) far from the surfaces of the spherical PE microdomains as depicted in Scheme 3. The increase in crowding effect of PCL subchains at the surfaces of 1D fibrous PE microdomains would result in the decrease in demixing degree of PCL subchains with epoxy matrix. Therefore, the *T*_g_’s of epoxy matrix increased. The fact that at the same composition the *T*_g_’s of epoxy matrices in the thermosets containing the fibrous PE microdomains were higher than those containing the spherical PE microdomains indicates that the crowding degrees of PCL at the surface of PE microdomains were dominant to affect the *T*_g_’s of epoxy matrices between the above two factors.

### 3.5. Toughening with PE Nanocrystals

**Figure 10:**
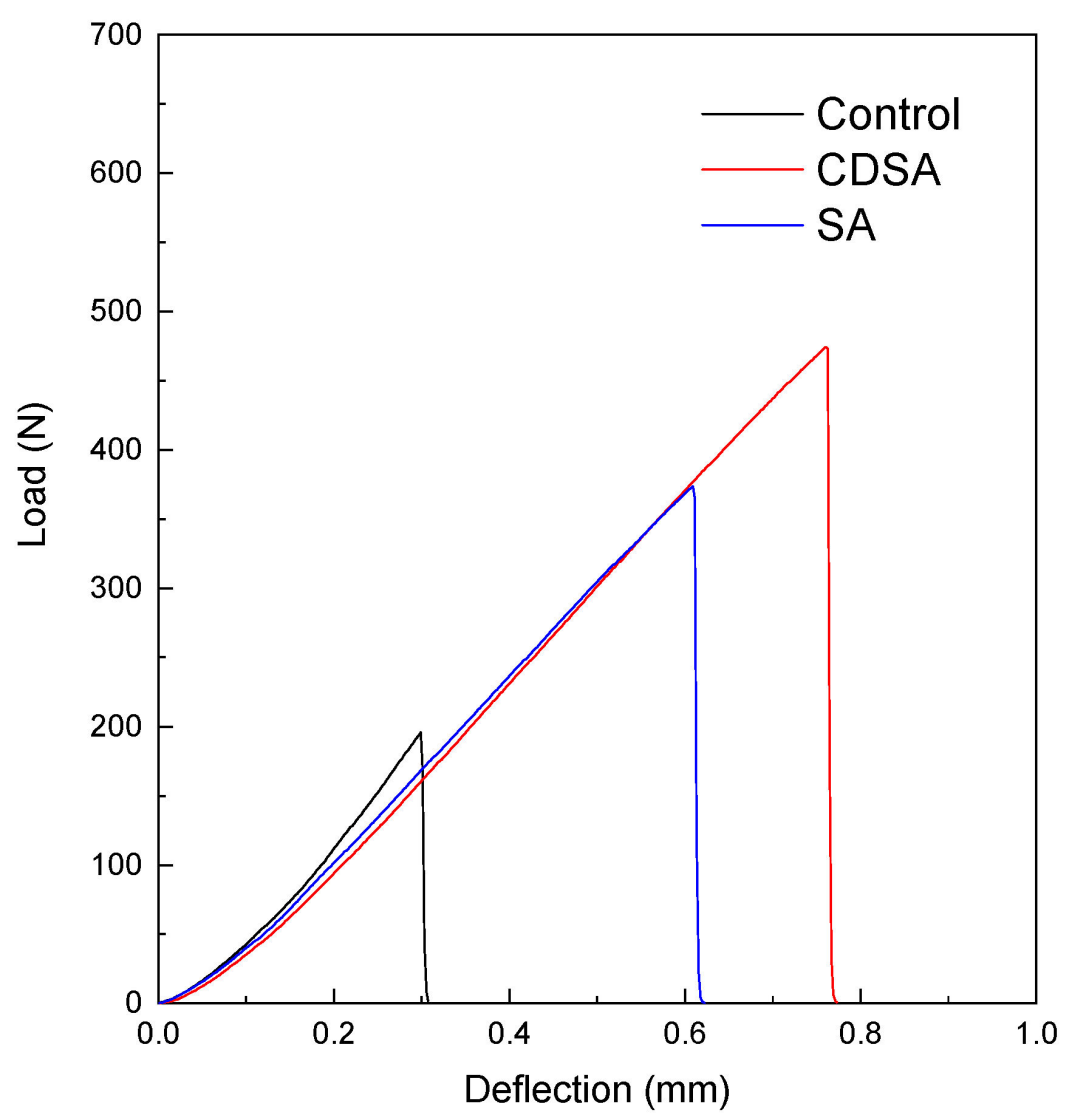
Load-deflection curves of control and the epoxy thermosets containing 10 wt% PCL*-b-*PE*-b-*PCL via CDSA or SA.

**Figure 11:**
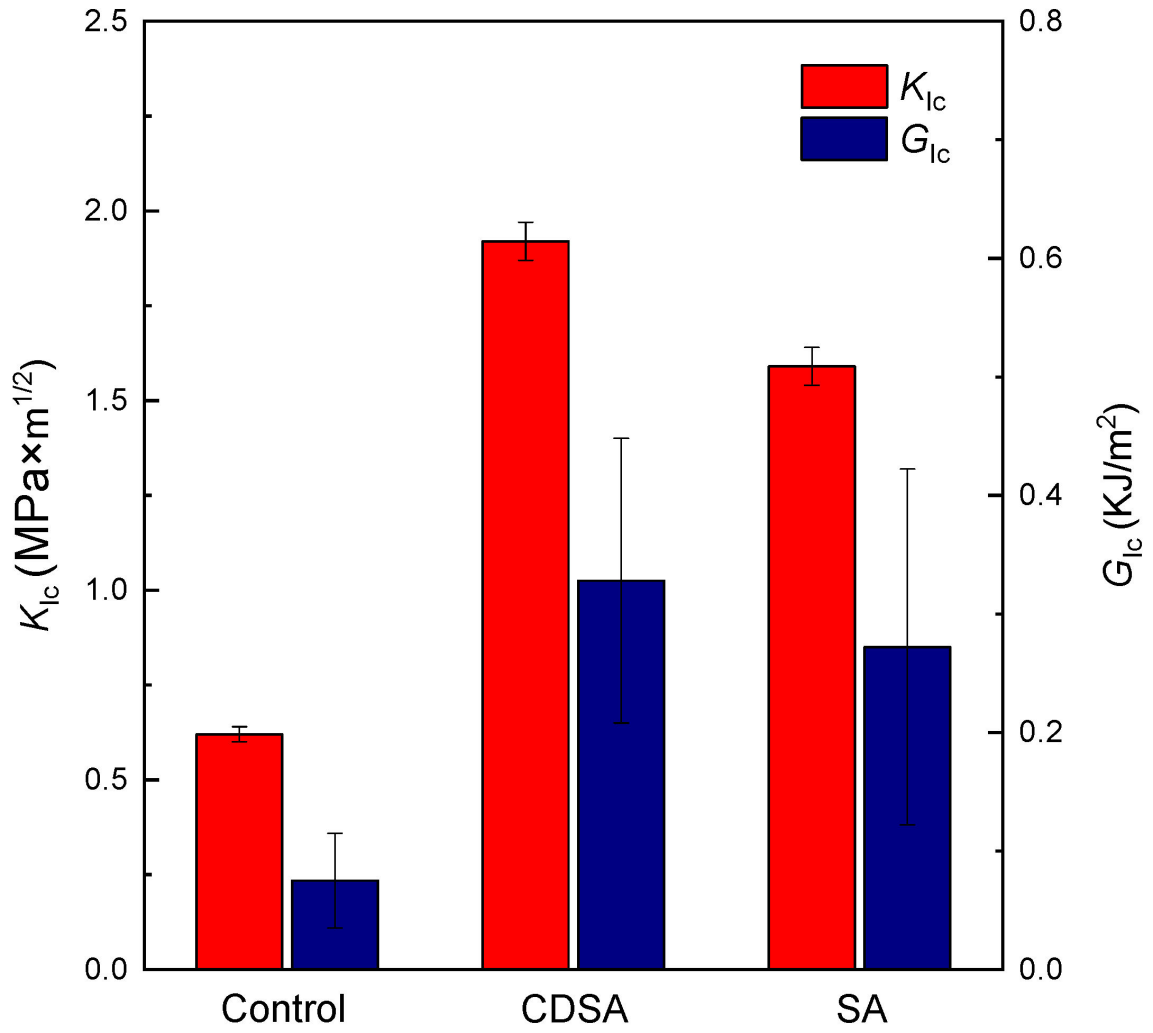
*K*_ⅠC_ values and *G*_ⅠC_ values of the control and nanostructured thermosets containing 10 wt% of PCL*-b-*PE*-b-*PCL via CDSA or SA.

The formation of PE nanocrystals reminds of investigating the fracture toughness of the materials. Herein, the critical stress field intensity factors (*K*_IC_’s) were measured via three-point bending tests (See Scheme S1). Shown in Figure 10 are the load-deflection curves for the measurements of critical stress field intensity factors (*K*_IC_); the related parameters were listed in Table S1. For the control epoxy, the load monotonously increased with the deflection, i.e., no yield point was exhibited. The specimen was broken with a small critical deflection at break (about 0.3 mm), suggesting that the control thermoset was quite brittle. Upon introducing 10 wt% of PCL*-b-*PE*-b-*PCL, notably, both critical load and deflection at break were increased, implying that the thermoset was significantly toughened. For the control epoxy, the *K*_IC_ and *G*_IC_ values were measured to be 0.62 MPa × m^1/2^ and 0.075 KJ/m^2^, respectively (See Scheme S1). For the nanostructured thermosets, the *K*_IC_ values were twice (viz. 1.59 MPa × m^1/2^) and thrice (1.92 MPa × m^1/2^) as that of control epoxy, the *G*_IC_ values were thrice (viz. 0.272 KJ/m^2^) and fourfold (0.328 KJ/m^2^) compared with that of control epoxy. The *K*_IC_ results indicate that the generation of PE nanocrystals resulted in the improvement in the fracture toughness. For the nanostructured thermosets by the use of BCPs, the improvement of toughness is attributable to the following mechanisms: (i) nanocavitation; (ii) plastic deformation of thermosetting matrix and (iii) the debonding of nanodomains out of microdomains [10,11,12,59]. In the present case, the rigid PE nanocrystals were incorporated into epoxy matrices. The nanocavitation would not be induced via the deformation of PE nanocrystals and thus could not be a vital toughening mechanism. In fact, the load-deflection curves did not display any yielding point, suggesting that no significant plastic deformation occurred in the thermosets under the present strain conditions. Therefore, the plastic deformation also was not the major cause of the toughness improvement. It is plausible to propose that in the present case, the improvement of toughness is attributable to the debonding of PE nanocrystals out of the epoxy matrices while the fracture occurred. It is of interest to note that the sample containing the 1D PE fibrous nanocrystals displayed the *K*_IC_ and *G*_1C_ values much higher than that containing the spherical PE nanocrystals (See Figure 11). The additional increase in *K*_IC_ and *G*_1C_ values could be associated with the formation of the long and fibrous PE nanocrystals, which could effectively hinder the propagation of cracks while the fracture occurs. The similar case was also found for the nanostructured thermosets containing cylindrical (or worm-like) nanoobjects by Bates et al. [11,60]. It is of interest to study the toughening mechanisms in the nanostructured thermosets containing 1D fibrous nanocrystals in depth.

## 4. Conclusions

The PCL-*b*-PCOE-*b*-PCL triblock copolymer was synthesized via the ring-opening metathesis polymerization (ROMP) of COE with a 1,4-butene moiety-containing PCL as the chain transfer agent. The triblock was successfully hydrogenated into the corresponding PCL*-b-*PE*-b-*PCL triblock. The latter was successfully used to generate the nanostructures in epoxy thermosets. It was found that with a two-step curing at 80 °C and then at 150 °C, the nanostructured thermosets were obtained, in which the 1D fibrous PE nanocrystals were generated. In contrast, only the spherical PE microdomains were generated in the nanostructured thermosets while a one-step curing at 150 °C was performed. The generation of 1D PE nanocrystals in epoxy followed so-called crystallization-driven self-assembly mechanisms. Depending on the morphologies of PE nanocrystals, the nanostructured thermosets displayed quite different thermal and mechanical properties. More importantly, it was found that the nanostructured thermosets containing the 1D fibrous PE nanocrystals had the *K*_IC_ and *G*_1C_ values much higher that containing the spherical PE nanocrystals. The *K*_IC_ value was even three times as that of control epoxy.

## Data Availability

Not applicable.

## Supplementary Materials

The following supporting information can be downloaded at: https://www.mdpi.com/article/10.3390/polym14183921/s1, Figure S1. GPC curves of PCL-Vi-PCL and PCL-*b*-PCOE-*b*-PCL; Table S1. The parameters of three-bending tests for the thermosets containing 10 wt% of PCL-*b*-PE-*b*-PCL triblock copolymer; Scheme S1. Schematic diagram of three-point bending specimen for the measurement of critical stress intensity factor (*K*_IC_).
