# Supplementary material for: Generation of One-Dimensional Fibrous Polyethylene Nanocrystals in Epoxy Thermosets
Source: Polymers (Basel). 2022 Sep 19;14(18):3921. doi: 10.3390/polym14183921 (PMC9501422; doi:10.3390/polym14183921)
Supplement: Supplementary file 1 [file polymers-14-03921-s001.zip › polymers-1915596-supplementary.pdf]

SUPPORTING INFORMATION

Generation of One-Dimensional Fibrous Polyethylene Nanocrystals in Epoxy Thermosets

Honggang Mei, Huaming Wang, Lei Li and Sixun Zheng *

College of Chemistry and Chemical Engineering and the State Key Laboratory of Metal Matrix Composites,Shanghai Jiao Tong University, Shanghai 200240, China

* Corresponding author, email: *szheng@sjtu.edu.cn (S. Zheng)*

TABLE OF CONTENTS

1. Experimental……………………………………………………………..S-3

1.1 Synthesis of Poly(ε-caprolactone) Chain Transfer Agent (PCL-CTA)………... S-3

1.2 Synthesis of PCL-*b*-PCOE-*b*-PCL Triblock Copolymer………………………..S-3

1.3 Synthesis of PCL-*b*-PE-*b*-PCL Triblock Copolymer…………………..……..S-4

1.4 Preparation of Nanostructured Thermosets...…………………………………..S-4

2. Techniques and Measurements…………………………………………..….S-4

2.1 Nuclear Magnetic Resonance (NMR) Spectroscopy...………………………... S-4

2.2 Gel Permeation Chromatography (GPC)………………………………………..S-4

2.3 Fourier Transform Infrared (FTIR) Spectroscopy………………..…………..S-4

2.4 Differential Scanning Calorimetry (DSC)...……………………………………..S-5

2.5 Wide Angle X-ray Diffraction (WXRD)…………………………………….….S-5

2.6 Transmission Electron Microscopy (TEM)…………………………………..….S-5

2.7 Dynamic Mechanical Thermal Analysis (DMTA)………..……………….….S-5

2.8 Measurements of Fracture Toughness………………………………………..….S-6

3. Schemes…………………………………………………………………..S-7

4. Tables…………………………………………………………..…………..S-8

5. Figures…………………………………………………….……………..S-9

1. Experimental

1.1. Synthesis of Poly(ε-caprolactone) Chain Transfer Agent (PCL-CTA)

To a fume-dried flask, *cis*-2-butene-1,4-diol (0.220 g, 2.50 mmol) and CL (30.000 g, 262.8 mmol) were added. The mixture was purged with nitrogen for 40 min and then $Sn(Oct)_2$ (0.1 wt% with respect of CL) was added as the catalyst. The polymerization was carried out at 120 °C for 24 hours. Cooled down to room temperature, the polymerized product was dissolved with tetrahydrofuran (60 mL) and the solution was dropwise added to cold methanol (400 mL). The precipitates were dried *in vacuo* at 40 °C for 24 hours; the product (29.980 g, denoted PCL-Vi-PCL) was obtained with a 99.2% yield. GPC: M_n = 12,100 Da with M_w/M_n = 1.38.

1.2. Synthesis of PCL-b-PCOE-b-PCL Triblock Copolymer

To a flask, PCL-Vi-PCL (10.000 g, 0.83 mmol), COE (10.000 g, 90.9 mmol) and anhydrous THF (160 mL) were added. The mixture was bubbled with nitrogen for 30 min and then Grubbs 2nd catalyst (10 mg) was added. The ring-opening metathesis polymerization (ROMP) of COE was carried out at 40 °C for 8 hours. After that, ethyl vinyl ether (1.0 mL) was added to terminate the polymerization. Thereafter, the mixture was dropwise added to cold methanol (500 mL) to precipitate the polymer. After drying *in vacuo* at 40 °C for 12 hours, the product (19.720 g, denoted PCL-*b*-PCOE-*b*-PCL) was obtained with the yield of 98.6%. ^{1}H NMR ($CDCl_3$, ppm): 5.39 (*m*, 96H, -C***H***=C***H***-), 4.08 (*t*, 92H, -COOC$\boldsymbol{H}_2$-), 3.67 (*t*, 4H, -C$\boldsymbol{H}_2$OH) 2.33 (*t*, 92H, -C$\boldsymbol{H}_2$COO-) 1.98 (*m*, 192H, -C$\boldsymbol{H}_2$-CH=CH-), 1.67 (*m*, 184H, -$CH_2C\boldsymbol{H}_2CH_2C\boldsymbol{H}_2CH_2$-), 1.40 (*m*, 92H, -$CH_2CH_2C\boldsymbol{H}_2CH_2CH_2$-), 1.34 (*m*, 192H, -$C\boldsymbol{H}_2CH_2$-CH=CH-) and 1.30 (*m*, 192H, -$C\boldsymbol{H}_2CH_2CH_2$-CH=CH-). GPC: M_n = 23,800 Da with M_w/M_n = 1.81.

1.3. Synthesis of PCL-b-PE-b-PCL Triblock Copolymer

To a flask, PCL-*b*-PCOE-*b*-PCL (15.000 g), *p*-toluenesulfonyl hydrazide (25.395 g), tri-*n*-propylamine (19.532 g), 2,6-di-*tert*-butyl-p-cresol (0.200 g) and xylene (150

mL) were added. Heated up to 135 °C, the hydrogenation was performed under refluxing condition for 10 hours. After that, the reacted mixture was cooled to room temperature and dropped into a great amount of methanol (500 mL) to afford the precipitates. The product (denoted PCL-*b*-PE-*b*-PCL) was dried *in vacuo* at 60 °C for 24 hour and the yield was calculated as 90.8 %. ^{1}H NMR (toluene-d_8, ppm): 4.00 (*t*, 90H, -COOC$\boldsymbol{H}_2$-), 3.40 (*t*, 4H, -C$\boldsymbol{H}_2$OH), 2.13 (*t*, 90H, -C$\boldsymbol{H}_2$COO-), 1.54 (*m*, 180H, -CH_2C$\boldsymbol{H}_2$$CH_2C\boldsymbol{H}_2$$CH_2$-), 1.38 (*m*, 760H, -C$\boldsymbol{H}_2$- in PE), 1.18 (m, 90H, -$CH_2$$CH_2C\boldsymbol{H}_2$$CH_2$$CH_2$-). FTIR (cm^{-1}, KBr window)：1726 cm^{-1} (>C=O), 720 cm^{-1} (CH_2 in the PE crystalline). In terms of ^{1}HNMR spectroscopy, the molecular weight was estimated to be M_n = 20,600 Da

2. Techniques and Measurements

2.1. Nuclear Magnetic Resonance (NMR) Spectroscopy

The ^{1}H NMR spectroscopy was carried out on a Bruker AVANCE 500 (500 MHz) spectrometer. The PCL-*b*-PCOE-*b*-PCL sample dissolved in $CDCl_3$ was measured at 25 °C. The PCL-*b*-PE-*b*-PCL sample was dissolved in deuterium toluene (toluene-d_8) and the solution was measured at 25 °C. The values of ^{1}H NMR chemical shift are expressed with reference to the residual solvent peak at 7.26 ppm of $CHCl_3$ or 7.14 ppm of toluene.

2.2. Gel Permeation Chromatography (GPC)

The molecular weights of PCL-Vi-PCL and PCL-*b*-PCOE-*b*-PCL were measured on a HLC-8320GPC instrument equipped with TSK gel GMHXL column (7.8 ID × 30 CM, 9 um) and a reflect index detector at 40 °C. Tetrahydrofuran was used as the eluent at a flow rate of 1.0 mL × min^{-1}. The solutions were passed through a 0.22 μm PTFE filter prior to injection, and the molecular weight values were expressed relative to polystyrene standards.

2.3 Fourier Transform Infrared (FTIR) Spectroscopy

The FTIR spectroscopy was carried out on a Perkin Elmer 1000 Spectrometer at room temperature. The solutions of PCL-*b*-PCOE-*b*-PCL and PCL-*b*-PE-*b*-PCL dissolved in tetrahydrofuran and toluene were casted on the KBr windows. The film specimens were measured at the resolution of 2 cm^{-1} with 64 scans.

2.4 Differential Scanning Calorimetry (DSC)

The DSC measurements were performed on Perkin-Elmer Diamond differential scanning calorimeter, equipped with an intra-cooler. About 8~10 mg of sample was put in an aluminum pan and then subjected to DSC measurements under a nitrogen atmosphere. All the epoxy samples were heated up to 200 °C and held at this temperature for 2 min and then quenched to −20 °C. And PCL-*b*-PE-*b*-PCL sample were heated up to 200 °C and held at this temperature for 2 min and then quenched to −20 °C. The samples were heated from -20 to 200 °C at a heating rate of 20 °C × min^{-1} and then cooled at a cooling rate of 10 °C × min^{-1}.

2.5 Wide Angle X-ray Diffraction (WXRD)

The wide-angle X-ray diffraction (XRD) experiments were carried out on a Shimadzu XRD-6000 X-ray diffractometer with Cu Kα (λ = 0.154 nm) irradiation at 40 kV and 40 mA using a Ni filter. Data were recorded in the range of 2θ =5~40° at the scanning rate of 6.0° × min^{-1}, respectively.

2.6 Transmission Electron Microscopy (TEM)

The TEM measurements were carried out on a JEM-2100 transmission electron microscope (JEOL Ltd, Japan), which was operated at the acceleration voltage of 200 kV. The nanostructured thermosets were trimmed using an ultrathin microtome machine and the thickness of specimens is about 70 nm.

2.7 Dynamic Mechanical Thermal Analysis (DMTA)

The DMTA measurements were carried out on a TA Instruments DMA Q800 dynamic mechanical thermal analyzer (DMTA) in a single cantilever mode and with liquid nitrogen as the coolant. The frequency used was 1.0 Hz and the heating rate 3.0 °C/min. The specimen dimension was 25 × 3.0 × 1.0 mm^3. The experiments were carried out from −80 °C to 200 °C.

2.8 Measurements of Fracture Toughness

The fracture toughness tests were carried out with a three-point bending mode at the speed of 1.3 mm × min^{-1} (See Scheme S1). A V-notch was machined in the middle of each specimen and the specimens were measured with ASTM E399 standard. Before the measurements, all the specimens were annealed at 80 °C for 24 hours. The critical stress intensity factor (K_{IC}) was calculated according to the following equations:

$$K_{IC} = \frac{P_C S}{B W^{\frac{3}{2}} f\left(a/W\right)} \tag{S1}$$

$$f\left(a/W\right) = \frac{3\left(a/W\right)^{\frac{1}{2}}\left[1.99-\left(a/W\right)\left(1-a/W\right)\times\left(2.15-3.93\,a/W+2.7\,a/W^{2}\right)\right]}{2\left(1+2\,a/W\right)\left(1-a/W\right)^{\frac{3}{2}}} \tag{S2}$$

where P_c was the peak load; S was span length; B is thickness of specimen; W was width of specimen; a was depth of V-shaped notch. The critical fracture energy (G_{IC}) was calculated according to the following equation:

$$G_{IC} = (1-\gamma^2)K_{IC} / E \tag{S3}$$

Where γ was the Poisson's ratio epoxy resin, 0.038 [1]; E was the flexural modulus of specimens.

REFERENCE

1. A. Smith, S. J. Wilkinson, W.N. Reynolds, The elastic constants of some epoxy resins, J. Mater. Sci. 9 (1974) 547-550.

3. Schemes

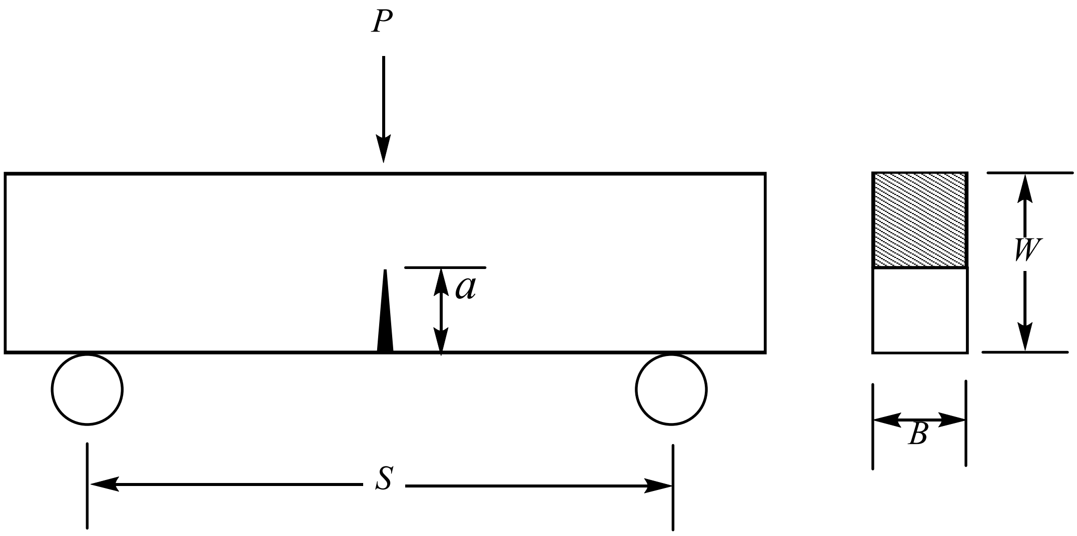

Scheme S1. Schematic diagram of three-point bending specimen for the measurement of critical stress intensity factor (K_{IC}).

4. Tables

Table S1. The parameters of three-bending tests for the thermosets containing 10 wt% of PCL-*b*-PE-*b*-PCL triblock copolymer.

Sample	P_c (N)	K_{IC} (MPa × $m^{1/2}$)	γ	E (GPa)	G_{IC} (KJ/m^2)
Control	195.96±20.1	0.62±0.02	0.38	6.95±0.8	0.745±0.04
CDSA	474.54±29.2	1.92±0.05	0.38	5.050±1.0	0.272±0.12
SA	373.32±31.3	1.59±0.05	0.38	5.010±1.0	0.328±0.15

5. Figures

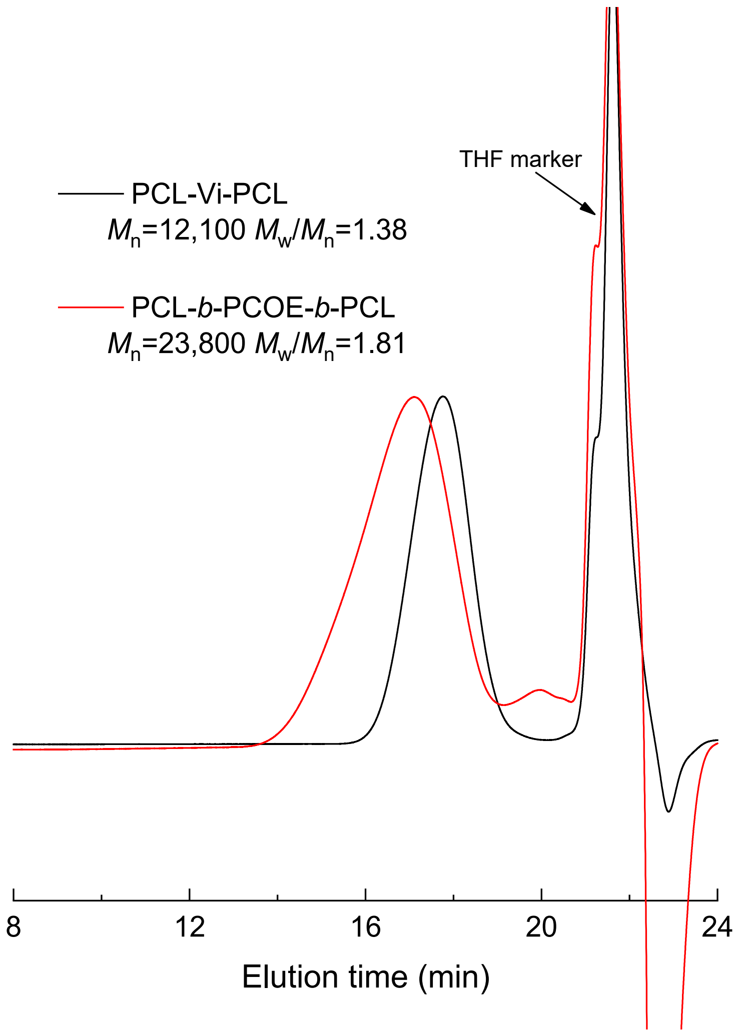

Figure S1. GPC curves of PCL-Vi-PCL and PCL-*b*-PCOE-*b*-PCL.
